# Genomics of the Uncultivated, Periodontitis-Associated Bacterium *Tannerella* sp. BU045 (Oral Taxon 808)

**DOI:** 10.1128/mSystems.00018-18

**Published:** 2018-06-05

**Authors:** Clifford J. Beall, Alisha G. Campbell, Ann L. Griffen, Mircea Podar, Eugene J. Leys

**Affiliations:** a Division of Biosciences, College of Dentistry, The Ohio State University, Columbus, Ohio, USA; b Biosciences Division, Oak Ridge National Laboratory, Oak Ridge, Tennessee, USA; c Genome Science and Technology, University of Tennessee, Knoxville, Tennessee, USA; d Division of Pediatric Dentistry, College of Dentistry, The Ohio State University, Columbus, Ohio, USA; University of Trento

**Keywords:** *Tannerella*, oral microbiology, periodontitis, single cell, WGA

## Abstract

Despite decades of research into the human oral microbiome, many species remain uncultivated. The technique of single-cell whole-genome amplification and sequencing provides a means of deriving genome sequences for species that can be informative on biological function and suggest pathways to cultivation. Tannerella forsythia has long been known to be highly associated with chronic periodontitis and to cause periodontitis-like symptoms in experimental animals, and *Tannerella* sp. BU045 (human oral taxon 808) is an uncultivated relative of this organism. In this work, we extend our previous sequencing of the *Tannerella* sp. BU063 (human oral taxon 286) genome by sequencing amplified genomes from 11 cells of *Tannerella* sp. BU045, including 3 genomes that are at least 90% complete. *Tannerella* sp. BU045 is more closely related to *Tannerella* sp. BU063 than to T. forsythia by gene content and average nucleotide identity. However, two independent data sets of association with periodontitis, one based on 16S rRNA gene abundance and the other based on gene expression in a metatranscriptomic data set, show that *Tannerella* sp. BU045 is more highly associated with disease than *Tannerella* sp. BU063. Comparative genomics shows genes and functions that are shared or unique to the different species, which may direct further research of the pathogenesis of chronic periodontitis.

**IMPORTANCE** Periodontitis (gum disease) affects 47% of adults over 30 in the United States (P. I. Eke, B. A. Dye, L. Wei, G. O. Thornton-Evans, R. J. Genco, et al., J Dent Res 91:914–920, 2012), and it cost between $39 and $396 billion worldwide in 2015 (A. J. Righolt, M. Jevdjevic, W. Marcenes, and S. Listl, J Dent Res, 17 January 2018, https://doi.org/10.1177/0022034517750572). Many bacteria associated with the disease are known only by the DNA sequence of their 16S rRNA gene. In this publication, amplification and sequencing of DNA from single bacterial cells are used to obtain nearly complete genomes of *Tannerella* sp. BU045, a species of bacteria that is more prevalent in patients with periodontitis than in healthy patients. Comparing the complete genome of this bacterium to genomes of related bacterial species will help to better understand periodontitis and may help to grow this organism in pure culture, which would allow a better understanding of its role in the mouth.

## Abstract (precis)

Periodontitis (gum disease) affects 47% of adults over 30 in the United States (P. I. Eke, B. A. Dye, L. Wei, G. O. Thornton-Evans, R. J. Genco, et al., J Dent Res 91:914–920, 2012), and it cost between $39 and $396 billion worldwide in 2015 (A. J. Righolt, M. Jevdjevic, W. Marcenes, and S. Listl, J Dent Res, 17 January 2018, https://doi.org/10.1177/0022034517750572). Many bacteria associated with the disease are known only by the DNA sequence of their 16S rRNA gene. In this publication, amplification and sequencing of DNA from single bacterial cells are used to obtain nearly complete genomes of *Tannerella* sp. BU045, a species of bacteria that is more prevalent in patients with periodontitis than in healthy patients. Comparing the complete genome of this bacterium to genomes of related bacterial species will help to better understand periodontitis and may help to grow this organism in pure culture, which would allow a better understanding of its role in the mouth.

## INTRODUCTION

The genus *Tannerella* comprises a set of bacterial species that have been found in the oral cavities of various mammals, including humans, cats (1, 2), dogs (3), and horses (4). The type species is Tannerella forsythia, (formerly known as Bacteroides forsythus and Tannerella forsythensis [5–7]). Additional related species have been identified by 16S rRNA gene sequencing, including the taxa designated *Tannerella* sp. BU063 (also known as human oral taxon 286 [HOT 286]) and *Tannerella* sp. BU045 (HOT 808) (8). We previously reported that several nearly complete genomes for BU063 (HOT 286) have been determined by single-bacterial-cell whole-genome amplification (WGA) and sequencing (9). Those BU063 genomes shared about 50% of their genes with T. forsythia strain 92A2 but had little synteny beyond operon level and were quite different in GC content (9). Recently, BU063 has been cultivated; however, it required proximity to other bacteria for efficient growth (10). The genome for cultivated BU063 (strain W11667) has recently been deposited in sequence databases (e.g., GenBank accession number CP017038).

Tannerella forsythia has long been known as a human periodontal pathogen, due to its increased abundance and prevalence in patients with chronic periodontitis (11, 12) and its ability to cause periodontitis-like symptoms in experimental animals (13, 14). Conversely, it does not appear to be associated with gingivitis or mild periodontitis in cats (1). A previous study from our group examined the prevalence of *Tannerella* sp. BU063 and T. forsythia by endpoint PCR of the internal transcribed spacer of the ribosomal operon using genus-specific primers (15). This work compared healthy subjects and patients with periodontitis and concluded that *Tannerella* sp. BU063 was found most often in healthy patients but that T. forsythia was found in patients with periodontitis. The methodology used for that study, however, was qualitative, and more-direct methods are now available to measure the abundance of specific bacteria.

Kistler and coworkers (16) identified an additional phylotype of *Tannerella* designated CP6_C2 or human oral taxon 916 by its 16S rRNA gene sequence (GenBank accession number KC203065), which is about 98% identical to both *Tannerella* sp. BU045 and *Tannerella* sp. BU063. Three additional genomes from gut microbes that are deposited in databases with the label *Tannerella* (i.e., “*Tannerella* CAG:118,” accession number CAYC010000000, “*Tannerella* CAG:51,” accession number CBHX000000000, and “*Tannerella* 6_1_58FAA_CT1,” GenBank accession number ACWX00000000) seem to be much more closely related to the genus *Coprobacter* based on BLAST analyses of 16S rRNA and other genes.

An earlier study had indicated that *Tannerella* sp. BU045 was highly associated with periodontitis (12). Therefore, it is of significant interest to determine the genome sequence of *Tannerella* sp. BU045 (HOT 808), as it may give insights into the disease process.

## RESULTS AND DISCUSSION

Sequencing libraries were prepared and sequenced from 11 bacterial single-cell amplified genomes (SAGs), numbered 101 to 111, and individual *de novo* assemblies were done (Table 1). SAGs 103, 108, and 110 represent relatively complete genomes (96.94%, 96.97%, and 89.87%, respectively, by CheckM [17]), suggesting that the genome size for *Tannerella* sp. BU045 is about 2.8 Mbp. All three genomes also contained at least 65 of a set of 66 core housekeeping genes (Table 1). Note that the GC content for *Tannerella* sp. BU045 is 56 to 58%, which is similar to the 55 to 56% GC content of BU063 (9) but substantially different from that of T. forsythia, at 47% GC.

**TABLE 1:** Characteristics of assemblies from *Tannerella* sp. BU045 single-cell amplified genomes 101 to 111

Assembly	*N*_50_	Total length	No. of contigs	Largest contig	GC content (%)	CheckM completeness (%)	No. of core genes (of 66)^a^
SAG 101	17,806	2,202,921	646	73,758	55.59	76.07	53
SAG 102	8,054	691,366	491	23,434	55.85	20.01	ND
SAG 103	18,376	2,779,294	921	84,200	56.77	96.94	65
SAG 104	9,600	1,565,841	870	34,268	56.31	43.10	ND
SAG 105	17,717	1,879,977	782	79,978	56.66	62.40	ND
SAG 106	11,176	9,329,179	4,175	68,043	56.05	76.11	ND
SAG 107	12,123	1,726,727	957	40,033	56.16	60.33	44
SAG 108	24,033	2,825,906	865	90,172	57.43	96.97	66
SAG 109	11,707	1,517,345	787	45,937	56.62	56.09	ND
SAG 110	23,317	2,797,228	855	82,488	56.50	89.87	65
SAG 111	11,725	1,869,816	712	46,230	56.99	70.03	42

a ND, not determined.

**FIG 1:**
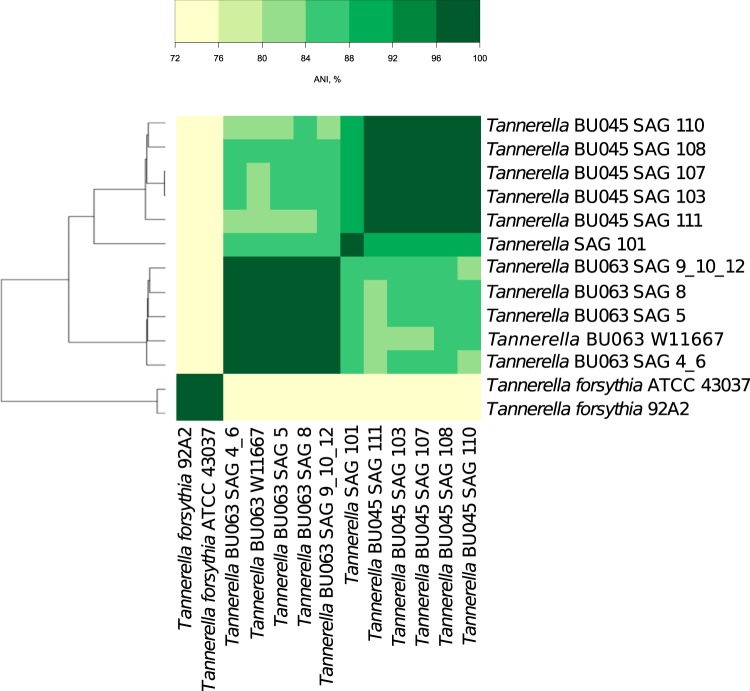
Heatmap of gANIs between various genome assemblies. Hierarchical clustering by the average method was performed using 100-ANI as the distance. Cells are colored as shown on the scale. Genomes along the vertical axis were the first in the comparison, and those on the horizontal axis were the second.

The pairwise genomic-average nucleotide identities (gANIs) and aligned fractions (AFs) for a set of *Tannerella* genomes (not including the putative *Coprobacter* genomes mentioned earlier) were determined. It was observed by the developers of the measurements that the usual thresholds for bacterial species are 96.5% for gANIs and 0.6 for AFs (18). Figure 1 shows a heatmap of gANIs between 6 assemblies of single cells from this study, 4 assemblies of *Tannerella* sp. BU063 cells from our previous study (9), the recently cultivated *Tannerella* sp. BU063 (W11667), and two Tannerella forsythia genomes. The T. forsythia genomes are for strain 92A2 (previously widely misidentified as strain ATCC 43037) and the true ATCC 43037 strain (19). Of the six newly sequenced BU045 single-cell genomes in Fig. 1, five of them formed a gANI clique that was over the 96.5% threshold for gANI (and the threshold of 0.6 for AFs, when incomplete assemblies were considered). We therefore consider these (SAGs 103, 107, 108, 110, and 111) as provisional conspecifics. A separate analysis using the mummer program (20) found that the five additional assemblies not included in Fig. 1 (SAGs 102, 104, 105, 106, and 109) also belonged to this ANI clique (over 96.5%), with SAGs 103, 104, 105, and 107 forming a nearly identical group (>99% ANI). It is possible that these four SAGs represent the same strain or closely related organisms. It is notable that they were derived from the same experimental subject. The set of nearly complete genomes, SAGs 103, 108, and 110, likely represent three different strains of the species.

The incomplete assembly from SAG 101 had gANI to the clique members of only 88 to 89% and may be derived from a different species, although the 16S rRNA gene extracted from the SAG 101 assembly has >99% sequence identity to *Tannerella* sp. BU045. Meanwhile, the *Tannerella* sp. BU063 SAGs and the cultivated W11667 strain had pairwise gANIs of over 96.5% within their group but gANIs to the BU045 group of 83 to 85%, and the Tannerella forsythia 92A2 and ATCC 43037 genomes had >96.5% gANI to each other but 72 to 74% gANI to all of the other compared genomes. Overall, the results support the existence of at least three discrete species (Tannerella forsythia, *Tannerella* sp. BU045, and *Tannerella* sp. BU063) within the *Tannerella* genus, and it is possible that SAG 101 represents a fourth species.

**FIG 2:**
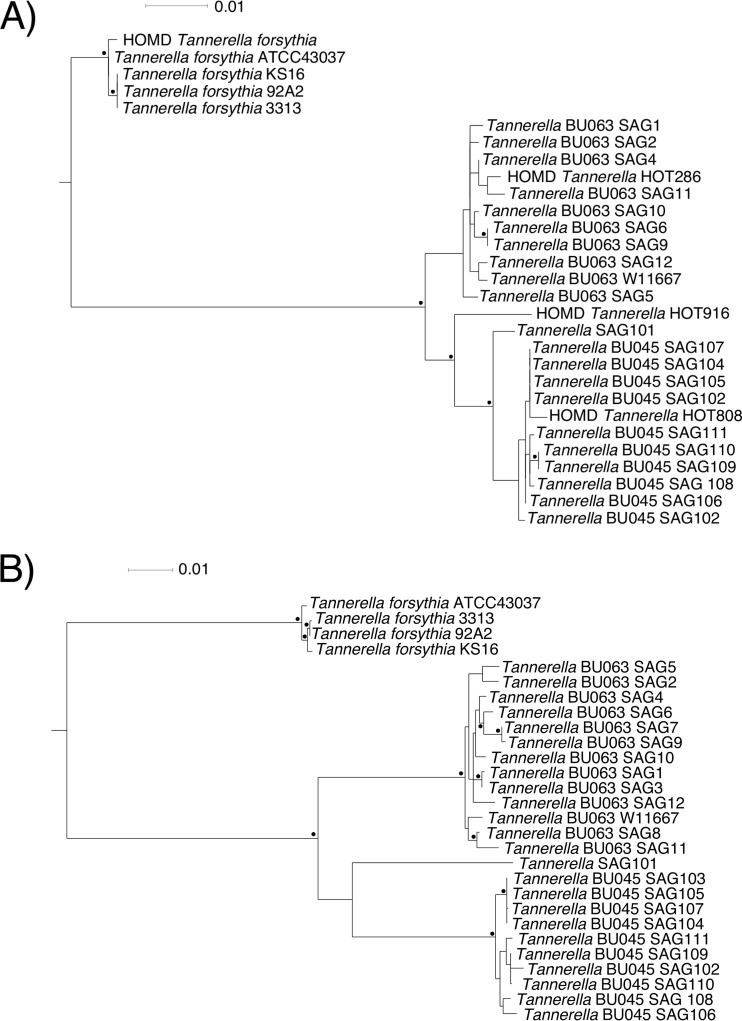
Maximum-likelihood phylogenetic trees of the *Tannerella* genus computed with 16S rRNA gene sequences (A) or concatenated protein sequences from 37 conserved genes (B). In both trees, Porphyromonas gingivalis W83 was used as an outgroup but was removed from the tree shown. The black dots represent branch points that are supported by bootstrap values of ≥70%. The scales represent 1% divergence. The nodes labeled “HOMD” in panel A are the representative sequences from that database. Other 16S rRNA gene sequences shown were extracted from the genomic contigs.

The relationship between genomes was confirmed by calculating maximum-likelihood phylogenetic trees using either 16S rRNA gene sequences or concatenated protein sequences from 37 genes that are highly conserved in bacteria (Fig. 2). These phylogenetic trees reinforce the finding that *Tannerella* sp. BU063 and BU045 are more related to each other than either is to T. forsythia. The newly cultivated BU063 strain W11667 exists in a well-supported clade with the previously sequenced BU063 SAGs, using either 16S rRNA or concatenated proteins. SAG 101 and *Tannerella* HOT 916/CP6_C2 appear to be closely related to BU045 but may represent separate species or subspecies. However, it is difficult to be certain about these relationships, since SAG 101 is a partial genome and there is only 16S rRNA gene information for HOT 916/CP6_C2.

**FIG 3:**
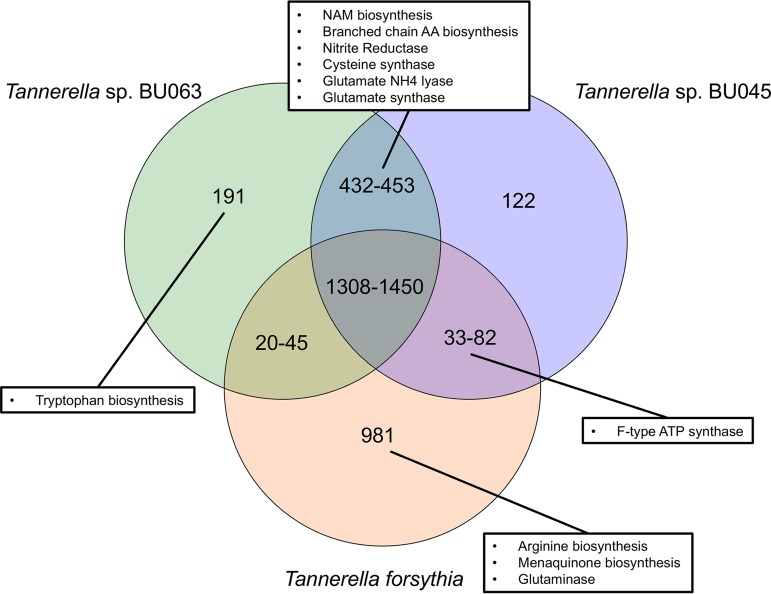
Venn diagram of core gene overlap of the *Tannerella* species. The presence of genes was evaluated using the “Profile and Alignment” tool on the IMG/ER website using thresholds of 50% identity and an E value of 10^−5^. Genes that are present in all genomes of each species were saved as a set, put into the “gene cart,” and evaluated for their presence in the genomes of the other species. Selected functional pathways that were found in the various categories are shown. NAM, *N*-acetylmuramic acid; AA, amino acid.

A core set of genes for each species was computed by starting with the genes from one strain and selecting the core genome that had homologs in other nearly complete genomes from that species (see Materials and Methods for details). The number of core genes was found to be 2,425 for T. forsythia, 2,214 for BU063, and 1,895 for BU045. The IMG/MER (21) Web tool was used to compare genes present in the core sets. However, it is notable that the numbers of shared genes determined varied slightly depending on the direction in which the analysis was done, i.e., depending on which core gene set was used to search for homologs. This may be due to duplicated or partial genes. Also, the single-cell genomes are likely to have missing regions, and sequencing or gene prediction errors may have occurred. Figure 3 presents a Venn diagram of shared and unique genes, with ranges representing uncertainty in the numbers calculated by different approaches. In Tables S1 to S5 in the supplemental material, we list the accession numbers of the genomes used, the gene identification numbers, and predicted products of the core gene sets from each species, and we indicate whether homologs were found in the genomes from the other two species.

10.1128/mSystems.00018-18.1 TABLE S1  Strain identifications or single-cell amplified genome (SAG) numbers, type of genome assembly, and accession numbers of the genomes used in the current study. Download TABLE S1, XLSX file, 0.01 MB. Copyright © 2018 Beall et al. 2018 Beall et al. This content is distributed under the terms of the Creative Commons Attribution 4.0 International license.

About one-half of the genes in each core genome are common to all three, while the distribution of the other categories generally reflects that BU045 contains fewer core genes than the other genomes and that BU045 and BU063 are closely related. There also is some indication of the BU045 assemblies being less complete, as seen by larger numbers of genes that are present in some but not all of the genomes in Table S1.

The pathway analysis tools in IMG/ER were used on the various groups of genes to make some predictions about the functional capabilities of the three species. First, although there is a set of genes unique to BU045, we were unable to ascribe well-defined functions to that group. In fact, 74 of the 122 genes were annotated as “hypothetical protein” or “protein of unknown function,” and many more had functions, such as restriction-modification, that are often found in mobile DNA (Table S4). T. forsythia 92A2, ATCC 43037, and BU063 shared 20 to 45 genes that were not found in BU045; however, none of these had well-defined metabolic functions. With regard to functions that were not ubiquitous in the genus, BU045 often resembled BU063. T. forsythia 92A2 and ATCC 43037 possessed pathways for arginine biosynthesis, glutaminase, and menaquinone biosynthesis that were lacking in both BU063 (9) and BU045. Similarly, several enzymes and pathways are present in both BU063 and BU045 but absent in T. forsythia, including the branched-chain amino acid biosynthesis pathway, cysteine synthase, glutamine synthase, nitrite reductase, and the pathway producing UDP-*N*-acetylmuramic acid from UDP-*N*-acetylglucosamine. The lack of the last pathway in T. forsythia is apparently why that organism requires exogenous *N*-acetylmuramic acid for growth (22). However, in two functions, BU045 resembled T. forsythia more than BU063. Genes predicted to allow the ability to biosynthesize tryptophan from chorismate are present in BU063 but absent in T. forsythia and BU045. Additionally, while all three species carry genes encoding vacuolar/archaeon-type rotary ATPase/ATP synthase subunits, BU045 and T. forsythia also carry genes for the subunits of the F_1_F_o_-type ATPase/ATP synthase, while BU063 lacks such genes (9).

Previously, it was examined whether genes that are associated with virulence in T. forsythia 92A2 were present in BU063 (9). A similar analysis was performed on BU045 genomes in the present study, using genes as predicted by IMG/ER and following up with tblastn analysis of the assembled contigs to find genes that might have been missed during annotation (Table 2). The distributions of such genes were identical between BU063 and BU045 except with the *wecC* gene, encoding UDP-*N*-acetyl-d-mannosaminuronic acid dehydrogenase. Homologs of *wecC* were found in some of the BU063 SAGs but not in the finished genome of the cultivated W11667 strain. This gene was also not found in any of the BU045 SAGs, although a homolog was found in SAG 101 (which, as mentioned earlier, may represent a different species). The *wecC* gene is involved in glycosylation of the S-layer and other extracellular proteins of T. forsythia (23), and a mutation in the gene affects biofilm formation (24) and T-helper-type 17 cell induction (25, 26). As seen in Table 2 (and reported previously for BU063 [9]), BU045 contained S-layer protein genes that were detectable by tblastn, although the regions of identity were sometimes fragmented in assemblies, possibly due to their lengths (3.5 to 4.3 kb).

**TABLE 2:** Virulence genes in Tannerella forsythia and their presence or absence in uncultivated genomes

Gene	Function	Present in BU063?	Present in BU045?
*bspA*	Surface protein	No	No
*kly*	Metalloprotease (karilysin)	No	No
*mir*	Metalloprotease (mirolysin)	No	No
*nanH*	Sialidase	No	No
*tfsA*, *tfsB*	S-layer proteins	Yes	Yes
*prtH*	Protease	No	No
*wecC*	UDP-mannosaminuric acid dehydrogenase (glycosylation, biofilm)	Yes	No (except in SAG 101)
*mgsA*	Methylglyoxal production	Yes	Yes

Both BU063 and BU045 appear to lack homologs of the protease karilysin or mirolysin (Table 2). These metalloproteases have been shown to inactivate complement, resulting in the protection of T. forsythia ATCC 43037 (27, 28). Recently, they have been discussed as part of a group of encoded proteins termed KLIKK proteases (29). The tblastn program was used to search for similar proteins, and it was found that both BU063 and BU045 have genes about 60% similar to the KLIKK serine protease miropsin-1 and other genes about 30% similar to both miropsin-1 and miropsin-2. They also had some sequences about 30% similar to mirolysin, but these sequences were incomplete and represented either pseudogenes, misassemblies, or whole-genome amplification artifacts. The level of 30% identity is substantially lower than that seen for other orthologous genes between these species, so it appears that these may not be functionally identical, though they probably encode secreted proteases.

**FIG 4:**
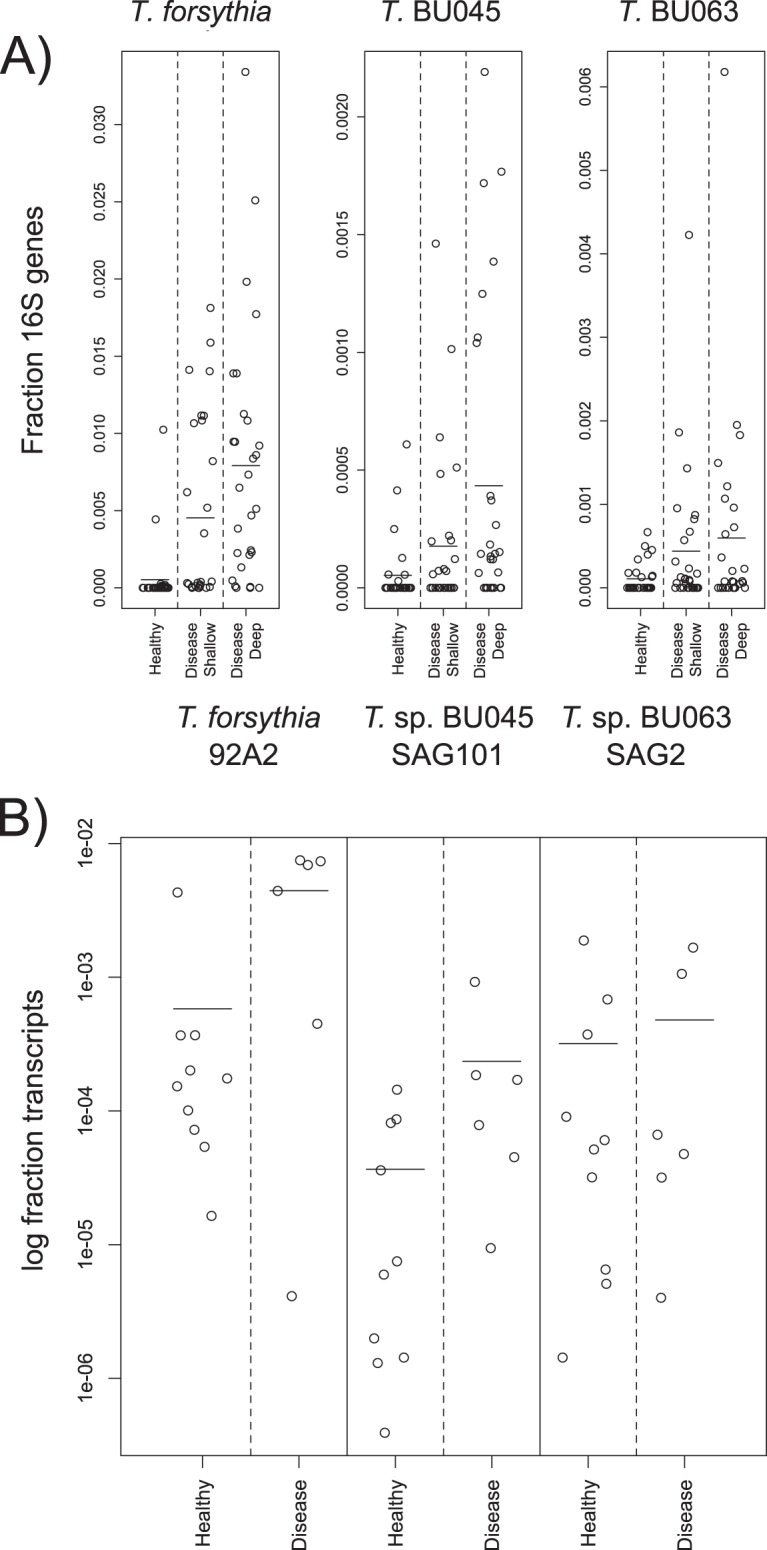
16S rRNA gene abundance and total gene expression of *Tannerella* species in healthy patient and periodontitis patient samples. (A) Fractional 16S rRNA gene abundances versus disease and periodontal pocket state for the 3 species. Data are from reference 12. Horizontal lines show the mean values. Wilcoxon rank sum test between healthy subjects and periodontitis patients (whose deep pockets were sampled). T. forsythia *P* = 1.01 × 10^–8^; *Tannerella* sp. BU045 *P* = 0.00039; *Tannerella* sp. BU063 *P* = 0.028. (B) Fraction of metatranscriptomic bacterial protein-coding gene expression for the species in samples from periodontitis patients and healthy controls. Raw data are from reference 30. Horizontal line segments indicate mean values. Wilcoxon rank sum tests: T. forsythia *P* = 0.0420; *Tannerella* sp. BU045 *P* = 0.0312; *Tannerella* sp. BU063 *P* = 0.875.

To examine the possible role of the *Tannerella* species T. forsythia, BU063, and BU045 in periodontitis, we reexamined two published data sets using our new genome sequences. One is 16S rRNA gene abundance data from our laboratory (12). This study used 29 healthy subjects and 29 patients with periodontitis, with the shallow and deep pockets of periodontitis patients sampled separately. Additionally, the newly derived genome assemblies were used to analyze metatranscriptomic data from a recent publication (30). That study used 10 healthy subjects and 6 patients with periodontitis. Those data were analyzed as discussed in Materials and Methods. The strains of the genomes used as mapping templates are shown in Fig. 4 and in Materials and Methods, although the metatranscriptomic reads that are quantitated by mapping likely derive from novel strains. Figure 4 shows the results of the two analyses. As previously determined (12, 30), Tannerella forsythia is highly associated with periodontitis. *Tannerella* sp. BU045 is also significantly associated with disease, although both its measured 16S gene abundance (which is not an absolute quantification) and its gene expression are lower than those of T. forsythia. *Tannerella* sp. BU063 has an intermediate measured abundance and less association with disease, with the metatranscriptomic data not showing a significant difference and the 16S data having borderline significance. BU063 was not identified as periodontitis associated in the earlier study because of false-discovery rate correction (12). The current observations are somewhat in contrast to those of a previous study seeming to show that BU063 was strictly health related (15). This might be due to differences in the patient populations or methodology.

Overall, the presence or absence of known genes involved in pathogenic processes do not give great insight into mechanisms driving the greater association of BU045 than BU063 with periodontitis, as seen in Fig. 4. It is possible that the common association of BU045 and T. forsythia with infected pockets is due to ecological differences in inflamed sites rather than to direct virulence determinants. Speculatively, if tryptophan is limited in healthy sites but more plentiful in disease sites with proteolytic bacteria, that might give BU063 an advantage in healthy sites relative to BU045 and T. forsythia. Another possibility is that the driving force is the presence of the F_1_F_o_ ATP synthase, one of the few well-defined functional proteins that is common to T. forsythia and BU045 but absent in BU063. Intriguingly, a recent transposon-sequencing (Tn-Seq) study using a mouse abscess model of Aggregatibacter actinomycetemcomitans found that mutations of the F_1_F_o_ ATP synthase in this organism affect its fitness in a monoinfection or a coinfection with Streptococcus gordonii (31). Although it is possible that similar mechanisms are at work with the *Tannerella* species, it may be worth noting that A. actinomycetemcomitans does not have genes for the V/A-type ATP synthase, which are present in all the *Tannerella* species.

## MATERIALS AND METHODS

### Sampling and DNA amplification.

Clinical sampling was approved by the Institutional Review Board of the Ohio State University and Oak Ridge site-wide Institutional Review Board (ORSIRB) for the National Laboratory. SAGs 101 to 108 were derived from periodontitis patients at the Ohio State College of Dentistry, and SAGs 109 to 111 were from volunteers from Oak Ridge, TN.

Subgingival plaque was collected using paper points, single cells were isolated with flow cytometry, and genomic DNA was amplified with Phi29 DNA polymerase. Single-cell amplified genomes (SAGs) corresponding to *Tannerella* sp. BU045 were identified by PCR and direct sequencing of a fragment of the 16S rRNA gene. Detailed procedures are given in previous publications (32, 33).

### DNA sequencing.

Sequencing libraries were prepared from 100 ng of amplified DNA with the NEBNext Ultra library kit (New England Biolabs, Ipswich, MA) and sequenced with 150-bp paired-end reads on the HiSeq 2500 sequencer (Illumina, San Diego, CA), producing 13.5 to 25.8 million reads per sample.

### Bioinformatics assembly and annotation.

The sequence reads were trimmed with Trimmomatic version 0.35 (34) using settings Illuminaclip 2:30:10:1:true, sliding window 4:15, and minlen 50. They were then assembled with SPAdes version 3.5 (35) in single-cell mode with otherwise-default parameters. The assemblies were evaluated with Quast 3.0 (36) and CheckM 1.0.5 (17).

Multiple approaches were used to search for and remove possible contamination of the genomes. RNAmmer (37) was employed to identify assembled contigs containing rRNA genes. The rRNA-containing contigs were used for a BLAST (38) search of the NCBI refseq_genomic database, restricted to bacterial sequences. If the 16S rRNA gene matched with greater than 99% identity the known *Tannerella* BU045 sequence, the contigs were assigned to a white list to ensure their inclusion in the final assembly. If they corresponded to unrelated bacteria, representative genomes were downloaded to act as BLAST databases to identify additional contaminating contigs. By this procedure, we identified a number of potential contaminants. rRNA genes similar to those from RefSeq accession number NZ_ACYI00000000.1 were found in SAGs 102 and 106. This genome currently is listed as “Enhydrobacter aerosaccus” in the NCBI, but the rRNAs appear to be extremely similar to those of Moraxella osloensis, a known oral community member only distantly related to the true Enhydrobacter bacterium (39). The assembled genome from SAG 110 contained contigs with ribosomal genes related to RefSeq accession number NZ_AOTF00000000.1, SR1 bacterium MGEHA (33). These genomes were used as search databases for the corresponding assemblies. The identified SAG assemblies were used as queries against BLAST databases of those genomes, while all assembled SAGs were used as queries against human genomic DNA, Saccharomyces cerevisiae (nuclear and mitochondrial genomes), Escherichia coli, phage PhiX 174, and the UniVec vector database. The search conditions used were “-task megablast -evalue.01 -max_target_seqs 1.” We also searched for simple repeat sequences with the dustmasker command and parameter “-level 50.” We removed contigs that were not white-listed rRNA-containing fragments and either matched the genomic sequences of over half the contig length (or over 500 bp) or matched UniVec sequences of over 48 bp. Since UniVec is not an exhaustive catalog of vector sequences, even short matches are likely to be problematic. Contigs that had greater than 75% of their length marked by dustmasker were likewise discarded.

The 3 assemblies that were close to full length, SAGs 103, 108, and 110, were uploaded to the IMG/MER website (21), and annotation was carried out by the default pipeline. Further screening for contamination was performed by examining contigs that contained unusual GC contents (<40%, visualized in the chromosome viewer of the IMG/MER website), unusual kmer content as visualized by principal-component analysis (PCoA) (Scaffold consistency [kmer frequency tool on the IMG/MER website]), or sequence similarity of >90% to genes from organisms outside the *Bacteroidetes* phylum (phylogenetic profiler on the IMG/MER website). As had been found for *Tannerella* sp. BU063, the gene annotation pipeline in IMG/MER misannotated a number of ribosomal protein genes that could be identified based on genome arrangement and protein similarity, so these annotations were manually corrected.

### Bioinformatics metatranscriptomics.

Metatranscriptomic raw data files were downloaded from MG-RAST (30, 40) for 10 samples from control subjects and 6 samples from patients with periodontitis. The bowtie2-build program was used to build a database from the combined genomes of Tannerella forsythia 92A2 (GCF_000238215.1) *Tannerella* sp. BU063 (GCF_000510385.1), and *Tannerella* sp. BU045 SAG 110 from this study. Reads were mapped to the genomes with bowtie2 2.2.6 using the parameters --very-sensitive-local, --no-unal, -X 1000, --score-min G,20,28, and --no-mixed. We used samtools 0.1.19 to convert the output to bam, sort, and index and samtools with gawk to remove reads that mapped in the ribosomal rRNA operons, as these had potential cross-reaction with other species. Finally, samtools idxstats was used to count the number of reads mapping to each genome.

To estimate the total number of bacterial-protein-coding reads from each sample, the raw reads were trimmed with Trimmomatic and reservoir sampling was used to select 50,000 random trimmed reads from each sample. We then mapped the protein-coding sequences of the sampled forward reads against the NCBI nr database (20 June 2015 version) using the Diamond program, version 0.8.14.76. (41) The alignments were then processed with the Meganize DAA File option of MEGAN community edition version 6.4.15 (42), and the total number of bacterial reads was estimated as the sum of bacterial reads in MEGAN divided by the sample size, 50,000, multiplied by the total number of reads per sample.

### Bioinformatics genome comparisons.

The genomic average nucleotide identity (gANI) was calculated by the method derived by Varghese and coworkers (18) as implemented on the IMG/ER website (Compare Genomes/Avg Nucleotide Ident./Pairwise ANI) (21). In this method, all protein-coding genes over 70% identical between two genomes are aligned and used to compute an average identity. Additionally, the method computes the aligned fraction (AF) of the two genomes. The numbers vary slightly depending on which genome is given first and which second. For the heatmap, genomes were clustered based on the percent nucleotide difference from this measurement using the R function hclust and the average method, also known as unweighted pair group using average linkages (UPGMA).

16S rRNA gene sequences were extracted from genome assemblies using RNAmmer (37) and aligned with SSU-ALIGN 0.1 (43), and masked alignments were converted from Stockholm format to Phylip with BioPython AlignIO (44). Concatenated protein alignments were generated using phylosift 1.0.1 with the command phylosift all-isolate -besthit (45, 46). Alignments were inspected, trimmed (to the equivalent of E. coli positions 29 and 1389 for 16S rRNA), and adjusted if necessary, and formats were changed using Mesquite 3.02 (47). Trees were calculated using RAxML (48) on the CIPRES computing cluster with Porphyromonas gingivalis W83 as an outgroup.

The set of core genes for each species was calculated with the phylogenetic Profiler Tool on IMG/ER (Find Genes/Phylogenetic Profilers/Single Genes) set to find genes in a template genome, plus 100% of the remaining genomes with a parameter E value of 10^−5^ and 50% identity. The gene sets used were (i) Tannerella forsythia 92A2 compared against Tannerella forsythia ATCC 43037, (ii) *Tannerella* sp. BU063 W11667 compared against *Tannerella* sp. BU063 SAG 2 and SAG 5, and (iii) *Tannerella* sp. BU045 SAG 108 compared against SAGs 103 and 110. We then compared the three core genome gene sets against the set of remaining genomes using the profile and alignment tool available in the gene cart area, again with the following settings: an E value of 10^−5^ and 50% identity. The resulting list was parsed to predict the number of genes that are shared between species. The tables in the supplemental material contain listings of the genomes used (Table S1), genes of core sets with indications of homologs in other species (Tables S2 to S4), and summary numbers (Table S5).

10.1128/mSystems.00018-18.2 TABLE S2  Presence of core genes from Tannerella forsythia in the genomes of the other *Tannerella* species. The genes are identified by their IMG/MER gene identifiers for the 92A2 genome and had homologs in the ATCC 43037 genome. The presence of homologs to these core genes is shown for the following genomes: *Tannerella* sp. BU045 (SAG 103), *Tannerella* sp. BU045 (SAG 108), *Tannerella* sp. BU045 (SAG 110), *Tannerella* sp. BU063 (SAG 2), *Tannerella* sp. BU063 (SAG 5), and *Tannerella* sp. BU063 (W11667). “1” indicates that a homolog was present and “0” that it was absent. Summary columns indicate the numbers of genomes with homologs from each species and totals. Download TABLE S2, XLSX file, 0.1 MB. Copyright © 2018 Beall et al. 2018 Beall et al. This content is distributed under the terms of the Creative Commons Attribution 4.0 International license.

10.1128/mSystems.00018-18.3 TABLE S3  Presence of core genes from *Tannerella* sp. BU063 in the genomes of the other *Tannerella* species. The genes are identified by their IMG/MER gene identifiers for the W11667 genome and had homologs in the SAG 2 and SAG 5 genomes. The presence of homologs to these core genes is shown for the following genomes: *Tannerella* sp. BU045 (SAG 103), *Tannerella* sp. BU045 (SAG 108), *Tannerella* sp. BU045 (SAG 110), Tannerella forsythia 92A2, and Tannerella forsythia ATCC 43037. “1” indicates that a homolog was present and “0” that it was absent. Summary columns indicate the numbers of genomes with homologs from each species and totals. Download TABLE S3, XLSX file, 0.1 MB. Copyright © 2018 Beall et al. 2018 Beall et al. This content is distributed under the terms of the Creative Commons Attribution 4.0 International license.

10.1128/mSystems.00018-18.4 TABLE S4  Presence of core genes from *Tannerella* sp. BU045 in the genomes of the other *Tannerella* species. The genes are identified by their IMG/MER gene identifiers for the SAG 108 genome and had homologs in the SAG 103 and SAG 110 genomes. The presence of homologs to these core genes is shown for the following genomes: Tannerella forsythia ATCC 43037, Tannerella forsythia 92A2, *Tannerella* sp. BU063 (SAG 2), *Tannerella* sp. BU063 (SAG 5), and *Tannerella* sp. BU063 (W11667). “1” indicates that a homolog was present and “0” that it was absent. Summary columns indicate the numbers of genomes with homologs from each species and totals. Download TABLE S4, XLSX file, 0.1 MB. Copyright © 2018 Beall et al. 2018 Beall et al. This content is distributed under the terms of the Creative Commons Attribution 4.0 International license.

10.1128/mSystems.00018-18.5 TABLE S5  Summary of core gene presence in other genomes. For each of the three core genome gene sets, the numbers of genes with homologs in different numbers of genomes of the other *Tannerella* species are tabulated. For instance, 981 Tannerella forsythia core genes had homologs in zero BU063 genomes and zero BU045 genomes, while 1,136 genes had homologs in all three BU063 and all three BU045 genomes. Download TABLE S5, XLSX file, 0.01 MB. Copyright © 2018 Beall et al. 2018 Beall et al. This content is distributed under the terms of the Creative Commons Attribution 4.0 International license.

### Data availability.

The data for SAGs 103, 108, and 110 have been deposited under NCBI BioProject number PRJNA342492, including SRA experiments SRX2157181, SRX2157182, and SRX2157183, and WGS accession numbers MIQB00000000, MIQC00000000, and MIQD00000000.

Software scripts used can be accessed at the OSU code repository with the URL https://code.osu.edu/beall-3/Single_Cell_Genomics.
